# Calorie restriction slows age-related microbiota changes in an Alzheimer’s disease model in female mice

**DOI:** 10.1038/s41598-019-54187-x

**Published:** 2019-11-29

**Authors:** Laura M. Cox, Marissa J. Schafer, Jiho Sohn, Julia Vincentini, Howard L. Weiner, Stephen D. Ginsberg, Martin J. Blaser

**Affiliations:** 1 Ann Romney Center for Neurologic Diseases, Brigham & Women’s Hospital, Harvard Medical School, Boston, MA USA; 2 Department of Medicine, NYU Langone Medical Center, New York, NY USA; 3 Cellular and Molecular Biology Training Program, NYU Langone Medical Center, New York, NY USA; 4 Psychiatry, Neuroscience & Physiology & the NYU Neuroscience Institute, NYU Langone Medical Center, New York, NY USA; 5 Center for Dementia Research, Nathan Kline Institute, Orangeburg, NY USA; 6 Department of Physical Medicine and Rehabilitation and Robert and Arlene Kogod Center on Aging, Mayo Clinic, Rochester, MN US; 7 Jacobs School of Medicine and Biomedical Sciences, University at Buffalo, State University of New York, Buffalo, NY USA; 8 Ecole Polytechnique Fédérale de Lausanne (EPFL), Lausanne, Switzerland; 9 Center for Advanced Biotechnology and Medicine, Rutgers University, New Brunswick, NJ USA

**Keywords:** Alzheimer's disease, Microbiome

## Abstract

Alzheimer’s disease (AD) affects an estimated 5.8 million Americans, and advanced age is the greatest risk factor. AD patients have altered intestinal microbiota. Accordingly, depleting intestinal microbiota in AD animal models reduces amyloid-beta (Aβ) plaque deposition. Age-related changes in the microbiota contribute to immunologic and physiologic decline. Translationally relevant dietary manipulations may be an effective approach to slow microbiota changes during aging. We previously showed that calorie restriction (CR) reduced brain Aβ deposition in the well-established Tg2576 mouse model of AD. Presently, we investigated whether CR alters the microbiome during aging. We found that female Tg2576 mice have more substantial age-related microbiome changes compared to wildtype (WT) mice, including an increase in *Bacteroides*, which were normalized by CR. Specific gut microbiota changes were linked to Aβ levels, with greater effects in females than in males. In the gut, Tg2576 female mice had an enhanced intestinal inflammatory transcriptional profile, which was reversed by CR. Furthermore, we demonstrate that *Bacteroides* colonization exacerbates Aβ deposition, which may be a mechanism whereby the gut impacts AD pathogenesis. These results suggest that long-term CR may alter the gut environment and prevent the expansion of microbes that contribute to age-related cognitive decline.

## Introduction

The gut-brain axis is an integrated network in which the microbiota and central nervous system communicate via endocrine, immune, and neural signaling pathways^1^. Several translational studies show that transferring microbiota from patients with neurodevelopmental and neurological disorders including autism, multiple sclerosis, and Parkinson’s disease can influence behavior, motor dysfunction, and immune responses in relevant animal models^2–5^. These studies provide evidence that intestinal microbiota may play an etiologic role in diseases that emerge at differing points during the lifespan. Consistent with this notion, Alzheimer’s disease (AD) patients have altered gut microbiota compared to age-matched healthy subjects^6,7^. In established animal models of AD, depleting the microbiota either in germ-free or antibiotic-treated mice served as protection against the pathological hallmark amyloid-beta (Aβ) plaque deposition^8,9^.

While host genotypes influence AD risk, the most important risk factor is advanced age^10^. In older adults, the microbiota is less diverse^11^, and immunosenescence and age-related changes in host physiology can destabilize the microbiota^12,13^. An ‘aged’ microbiota promotes immune dysfunction, including increased systemic inflammation and impaired macrophage phagocytosis, which can be partially restored by transferring microbiota from young to aged mice^14^. Thus, understanding how to slow or reverse age-related changes in the gut microbiota has therapeutic implications for age-related brain diseases, including AD.

Diet is a major environmental factor that modulates the microbiota and has been proposed to prevent age-related changes of the microbiota^11^. Calorie restriction (CR), characterized by 20–40% reduction of total calorie intake without malnutrition, increases the healthspan and lifespan in multiple model organisms^15^. A 30% reduction in calories from carbohydrates activates neuroprotective signatures and suppresses age-related transcriptional changes in the hippocampus in wildtype (WT) mice^16^. In the context of AD, we found that CR prevents Aβ plaque accumulation and modulates the expression of the gamma-secretase complex, the amyloid-beta precursor protein (APP) processing enzymes, in a sex-dependent manner in Tg2576 mice^17^. In addition to effects on host physiology, CR modulates the microbiota and increases abundances of bacteria that positively correlate with lifespan^18^. However, the association between CR, the microbiome, and AD pathogenesis has not been established.

In this study, we investigated the effect of long-term 30% CR compared with *ad libitum* (AL) feeding on the microbiome in aging. We studied the Tg2576 model, where a mutant variant of the human APP originally identified in a Swedish family with early-onset AD (APPswe) is expressed in transgenic mice^19,20^. This transgene results in cerebral amyloid accumulation, synaptic loss, and cognitive impairment by 12 months of age (MO)^20,21^. We now interrogate whether the CR effects on the intestinal microbiome in aging can be related to the susceptibility to pathologic lesions in the brain. We also investigated how the microbiota changed with age in WT littermates which do not develop Aβ pathology. This study demonstrates for the first time that female Tg2576 mice show enhancement of age-related microbiota changes compared to WT littermates, and that CR reverses age- and Aβ-related changes in the gut microbiota.

## Results

### Diet, APPswe, and sex shapes the microbiota in a model of AD

**Figure 1:**
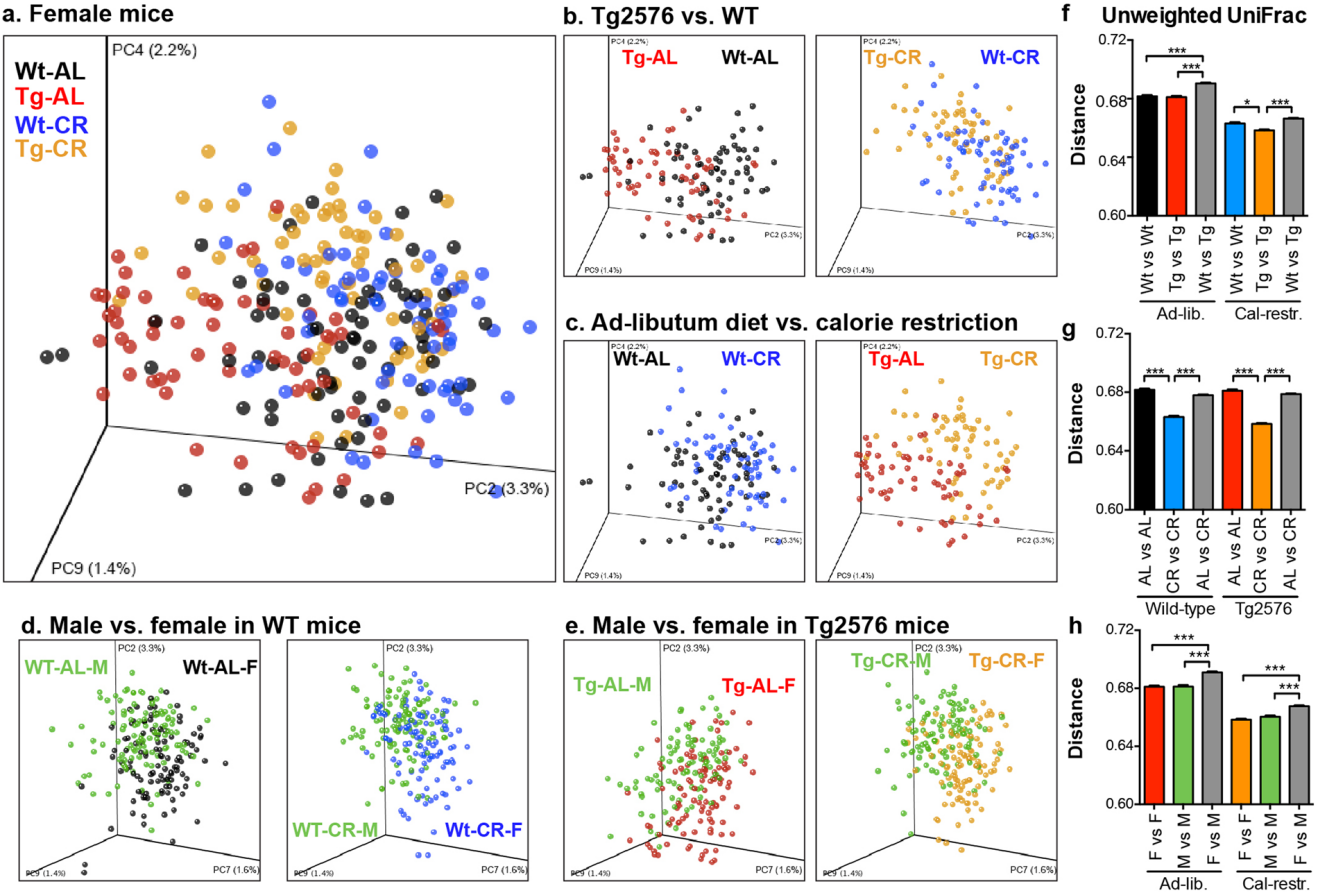
Genotype, diet, and sex shape microbiota structure. Differences between microbiota were visualized by PCoA of unweighted UniFrac distances **(**panels a–e). (**a**) Overall stratification by diet and mouse genotype. (**b**) Tg2576 mice show altered microbiota from littermate WT mice, regardless of diet. (**c**) Diet shifts the microbiota in both WT and Tg2576 mice. (**d**,**e**) Male and female mice show differences in microbiota. (**b**–**e**) Clusters were significantly different by Permanova test, p < 0.05. (**f**) Unweighted UniFrac distances between WT and Tg2576 female mice (gray bars) were larger than within genotype distances. (**g**) CR reduces intragroup microbiota variation compared to AL-fed mice. (**h**) UniFrac distances between males and females were larger than intragroup distances within each sex. Bonferroni adjusted t-test, *p < 0.05, ***p < 0.001.

We administered a 30% CR diet (with reduction in carbohydrates only) to male and female Tg2576 and nontransgenic WT littermates, initiating the diet at 2.5–3 MO and randomly assigning the diet without prior knowledge of microbiota composition. We sequenced the microbial 16S rRNA gene from longitudinally collected fecal samples at 11 different time points until mice reached 15 MO (n = 15–17 per group, with n = 7–9 per group followed until 15 MO). Differences in overall microbiota communities (β-diversity) were determined by comparing unweighted UniFrac distances. By Permanova testing, we found that microbiota communities differed significantly (p < 0.05) in mice by genotype, diet, and biologic sex **(**Fig. 1a–e and Supplementary Fig. 1**)**. In addition to three-dimensional visualization of community structure, pair-wise UniFrac distances were used to examine the overall dissimilarity of microbiota composition **(**Fig. 1f–h**)**. Differences between the groups (gray bars, e.g. WT mice versus Tg2576 mice stratified by sex and diet) were significantly larger than within-group differences (e.g., WT mice compared to other WT mice), indicating significant effects of genotype, diet, and sex on microbiota composition (Fig. 1f–h**)**. In addition, while both genotypes showed similar intra-group UniFrac distances **(**Fig. 1f**)**, CR-fed mice had reduced microbiota variation compared to AL-fed WT and Tg2576 mice, indicating that CR selects for a more homogeneous microbiota regardless of genotype.

Examining α-diversity over the course of the study, Tg2576 mice showed consistently lower phylogenetic diversity than WT mice at multiple time points **(**Supplemental Fig. 2), although overall changes were not statistically significant. Conversely, CR-fed mice showed slightly higher α-diversity. High α-diversity has been associated with health, whereas lower α-diversity has been associated with obesity^22^ and inflammatory bowel disease. Thus, while the effect sizes are small, CR appears to restore the α-diversity losses observed in Tg2576 female mice.

### The effect of CR on the microbiota in aging

**Figure 2:**
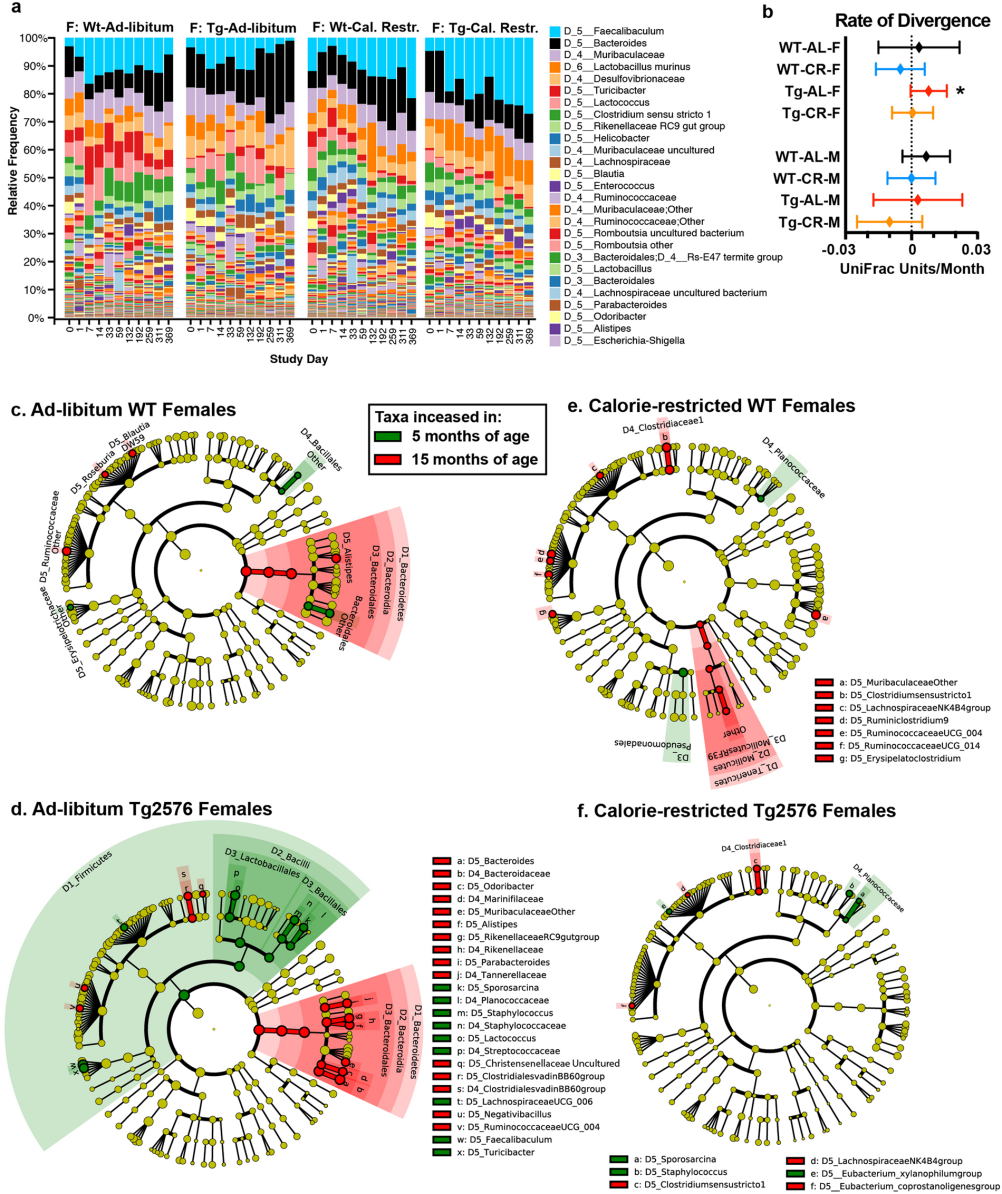
The effect of CR on the aging microbiota in WT and Tg2576 mice. **(a**) Composition of the fecal microbiota from study day 0 (~3 MO) until study day 369 (~15 MO). Mice were individually housed throughout the experiment, eliminating cage effects. (**b**) Age-related microbiota drift. Divergence from microbiota at study day 59 was measured using unweighted UniFrac distances on a per-mouse basis; slopes were calculated and tested by linear regression whether significantly non-zero. *p < 0.05. (**c**–**f**) Microbial taxa that are increased in 5 MO or 15 MO female mice, LEfSe p < 0.05. Each cladogram represents all taxa detected at >0.1%, shown at the Kingdom phylogenetic level through the genus level. A yellow circle depicts taxa present, but not enriched. Red circles are enriched in aging, and green enriched in young animals. The size of the circle corresponds to the population of each taxon.

Next, we examined whether there were any consistent age-related changes in the microbiota **(**Fig. 2**)**. Microbiota composition in females from 3 to 15 MO is shown in Fig. 2a. Mice were individually housed throughout the course of the study, eliminating cage effects, and thus we could measure microbiota drift in a single animal over time. To assess how the microbiota in a single mouse diverges as they age **(**Fig. 2b**)**, we calculated the unweighted UniFrac distances between the microbiota at study day 59 (5 MO) and later microbiota samples (7–15 MO) on a per-mouse basis. We selected study day 59 because there is a substantial weight loss during the initial 30 days of CR, followed by stabilization in weight. We found increased microbiota divergence in aging AL-fed Tg2576 mice (positive slope, significantly non-zero, linear regression), whereas CR-fed Tg2576 mice and all other groups did not show a significant increase in divergence over time.

To determine the basis for this variation, we identified specific taxa (at the phylum to genus level) that changed with aging **(**Fig. 2c–f**)**. In AL-fed females, we found that age increased the order *Bacteroidales* and genus *Alistipes* and decreased the order *Bacillales* compared to young mice **(**Fig. 2c,d). These changes occurred in both the WT and Tg2576 mice, indicating that these specific age-related microbiota changes are independent of APPswe expression. Similar increases in *Bacteroides* were found in humans in association with both aging and AD^6,13^. In AL-fed Tg2576 female mice, additional age-related changes not observed in WT littermates included decreases in the phylum *Firmicutes* and an increase in lower order taxa in the phylum *Bacteroidetes*. Genera within *Firmicutes* including *Faecalibaculum*, *Turicibacter, Lachnospiraceae, Lactococcus, Staphylococcus*, and *Sporosarcina* and genera within *Bacteroidetes* including *Bacteroides, Odoribacter and Parabacteroides* appeared to drive overall differences **(**Fig. 2d**)**. CR-fed mice had less substantial change in their microbiota than AL-fed mice **(**Fig. 2e,f**)**. With the exception of *Planococcaceae* and genus *Staphylococcus*, nearly all of the age-related microbiota differences observed in AL-fed females were rescued in the aged CR-fed mice. Both CR-fed WT and Tg2576 mice showed increased *Clostridium sensu stricto 1* and *Lachnospiraceae NK4B4* group in aging. In summary, these data indicate that in female mice, CR substantially and consistently reduces the age-related changes in the microbiota, especially in Tg2576 mice. We also identified specific taxa that changed during aging in male mice **(**Supplementary Fig. 3**)**. With aging, all males showed an increase in *Ruminococcaceae UCG_004* and *Lachnospiraceae NK4B4* group, and CR-fed males showed a decrease in *Bacteroides* and *Eubacterium xylanophilum*. However, all other changes were inconsistent between the 4 different groups, suggesting that the effect of aging and CR on the microbiota in male mice is less uniform than in females.

Protective effects of CR are sex-specific^17^. Moreover, sex-specific microbiota differences can influence chronic disease^23^. Therefore, we examined microbiota differences between males and females. Female mice have higher *Gammaproteobacteria* than males regardless of age, genotype, or diet **(**Supplementary Fig. 4**)**. With aging, there are broad phylum level changes in AL-fed mice, with higher *Proteobacteria* and lower *Firmicutes* in 15 MO WT and Tg2576 females compared to males. Aged AL-fed WT and Tg2576 females have higher *Bacteroides* and lower *Faecalibaculum* compared to males, but these sex-specific differences were not observed with CR. These data indicate that the age-related depletion in *Faecalibaculum* and increase in *Bacteroides* observed in females are not present in males and that CR can restore the levels of these microbes to those seen in WT aged males **(**Supplementary Fig. 4c**)**.

### Sex-specific associations between AD pathology and the microbiota in aging

**Figure 3:**
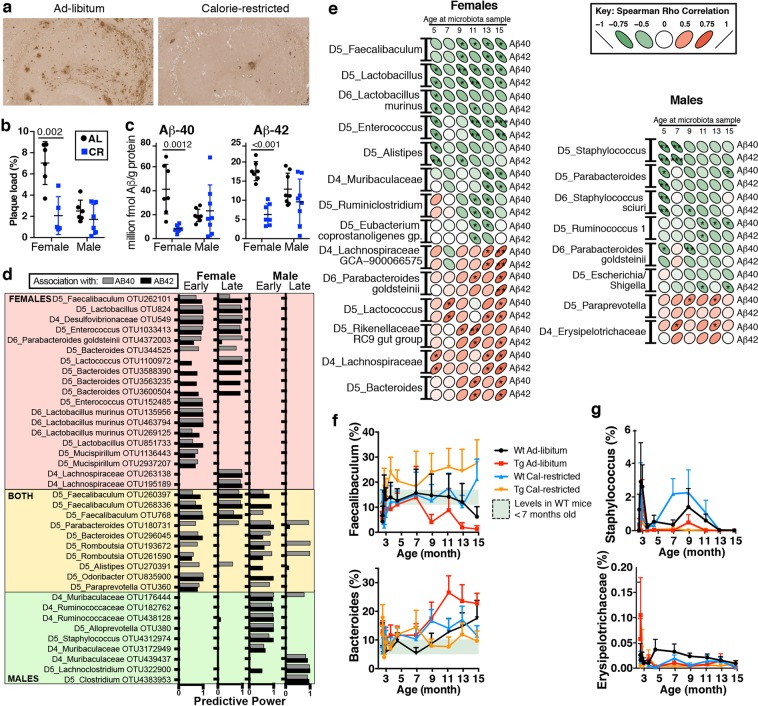
CR modulates plaque load and microbiota in a sex-specific manner. (**a**) Aβ40 and Aβ42 levels are reduced in the entorhinal cortex by CR (blue) in female Tg2576 mice, n = 7–8/group, T-test, as measured by ELISA (AL, black). (**b**) CR decreases Aβ plaque load in the hippocampus in females, as determined by immunohistochemistry. (**c**) Aβ40 and Aβ42 levels in the entorhinal cortex. Panels a–c modified from Schafer et al., Neurobiology of Aging 2015 with permission. (**d**) OTUs from younger (Early, 5–10 MO) or older (Late, 12–15 MO) mice, that were predictive of Aβ40 and Aβ 42 levels in the brain. (**e**) Microbiota that correlate with Aβ40 and Aβ42 levels differ by sex. *p < 0.05, ** < 0.01, *** < 0.001, Spearman correlation. (**f**) AL-fed Tg2576 female mice (red) show accelerated age-related changes in *Faecalibaculum* and *Bacteroides* compared to WT (black) and are reversed by CR (orange). (**g**) Males show minimal age-related bacterial patterns that correlate with Aβ plaque burden.

Investigating the presence of Aβ plaques in Tg2576 mice by histology and ELISA showed that CR significantly reduced Aβ deposition in the brains of female but not male mice (Fig. 3a–c)^17^. Because microbiota composition can drive sex-specific disease susceptibility^23,24^, we asked whether the protective effect of CR in female mice was related to the microbiota. We used a random forest test with the Boruta algorithm^25^ to identify microbiota operational taxonomic units (OTUs) associated with brain Aβ40 and Aβ42 levels. In female mice, *Faecalibaculum*, *Bacteroides, Lactobacillus*, *Enterococcus*, *Lactococcus, Parabacteroides goldsteinii*, and *Desulfovibrionaceae* were associated with Aβ40 or Aβ42 levels in the hippocampus **(**Fig. 3c**)**. Both males and females shared significantly associated OTUs belonging to *Faecalibaculum*, *Bacteroides, Romboutsia*, and *Parabacteroides*. In male mice, there were additional OTUs associated with Aβ, including those identified as *Ruminococcaceae*, *Alloprevotella*, *Staphylococcus*, *Lachnclostridium, Muribaculaceae*, and *Clostridium*. Several taxa from samples collected before the expected development of amyloid plaques, including *Faecalibaculum*, *Bacteroides*, *Enterococcus*, *Desufovibronaceae* and *Parabacteroides* were associated with Aβ, consistent with the hypothesis that changes in the microbiota that precede disease development may influence neuropathology.

To determine whether the direction of these relationships was direct or inverse, we correlated bacterial populations with Aβ40 and Aβ42 levels in the brain **(**Fig. 3d**)**. Results indicated several taxa were specifically linked with either low Aβ (green ellipse) or high Aβ (red ellipse). In females, *Faecalibaculum* and *Lactobacillus* had the strongest association with Aβ protection, and *Bacteroides* and *Lachnospiraceae* had the strongest association with elevated pathogenic Aβ levels.

We next asked whether bacteria associated with Aβ changed in abundance over time and whether this was affected by long-term CR. *Faecalibaculum* and *Bacteroides* were the two most abundant taxa in this study. Following stabilization on the control diet **(**Supplementary Fig. 5**)**, *Faecalibaculum* decreased in WT-AL mice from 13–15 MO **(**Fig. 3e**)**. Tg2576 mice had an accelerated age-related decrease in *Faecalibaculum*, starting at 9 MO with levels dropping 10-fold. However, in CR-fed Tg2576 female mice, the diet restored *Faecalibaculum* levels in aging above those observed in young WT mice. We also found that *Bacteroides* levels nearly doubled during aging in AL-fed WT females and more than doubled with age in Tg2576 mice. However, CR reduced *Bacteroides* in Tg2576 mice to levels observed in young WT mice. In humans, *Bacteroides* increase with aging^13^ and in AD^6^, and their toxins have been hypothesized to play a role in AD pathogenesis^26^. In male mice, the strongest correlations with Aβ levels were with *Staphylococcus* and *Erysipelotrichaceae*. These bacteria represented minor populations (<1% in Tg2576 mice) and did not show altered population levels with aging or with respect to diet **(**Fig. 3f**)**. In summary, CR suppresses age-related changes in the taxa that were linked with AD pathology in female mice.

### Microbiota predicted functions associated with AD pathology

**Figure 4:**
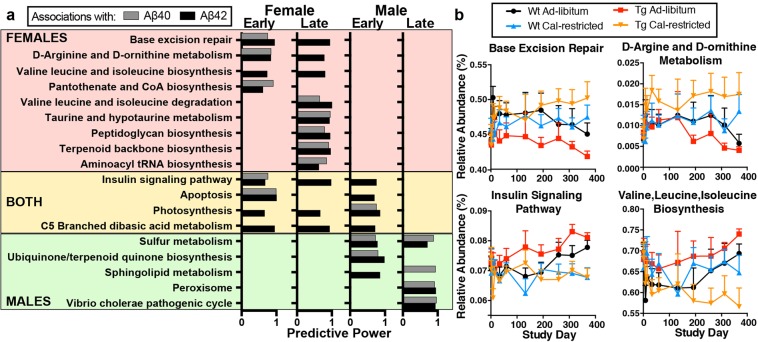
Bacterial KEGG Pathways associated with disease. (**a**) KEGG pathways from early (5–11 MO) or late (12–15 MO) that were predictive of Aβ40 and Aβ42 levels in the brain. (**b**) Relative abundance of select KEGG pathways over time.

To investigate microbial functional changes, we used PICRUSt^27^ to reconstruct a predicted microbial metagenome and we classified genes into KEGG orthologous groups (KOs) and pathways^28^. We found pathways involved in amino acid metabolism and tRNA biosynthesis linked with Aβ40 and Aβ42 levels in female Tg2576 mice **(**Fig. 4**)**. Specific amino acid metabolites produced by the intestinal microbiota can modulate immunity in the central nervous system^29,30^, thus the effect of CR on these putative functions may be important for AD. We also found base excision repair, pantothenate biosynthesis, peptidoglycan synthesis, and terpenoid biosynthesis linked with Aβ levels, implicating microbial DNA damage, vitamin production, immune signaling, and antioxidant production in the APPswe-related Aβ pathology. Both male and female mice showed associations between the insulin signaling pathway, apoptosis, photosynthesis, and dibasic amino acid metabolism and Aβ pathology. In male mice, we found an association with sulfur, terpenoid, and sphingolipid metabolism, the peroxisome pathway, and *Vibrio cholerae* pathogenic cycle, suggesting that proinflammatory mediators, or changes in lipid or antioxidant metabolism could shape the Aβ response, as opposed to protein metabolism which was detected in females. Paralleling the changes in specific microbes, we found that Tg2576 mice show accelerated age-related changes in microbiota functions, which were reversed by CR.

### CR modulates intestinal transcriptional signatures in aged female mice

**Figure 5:**
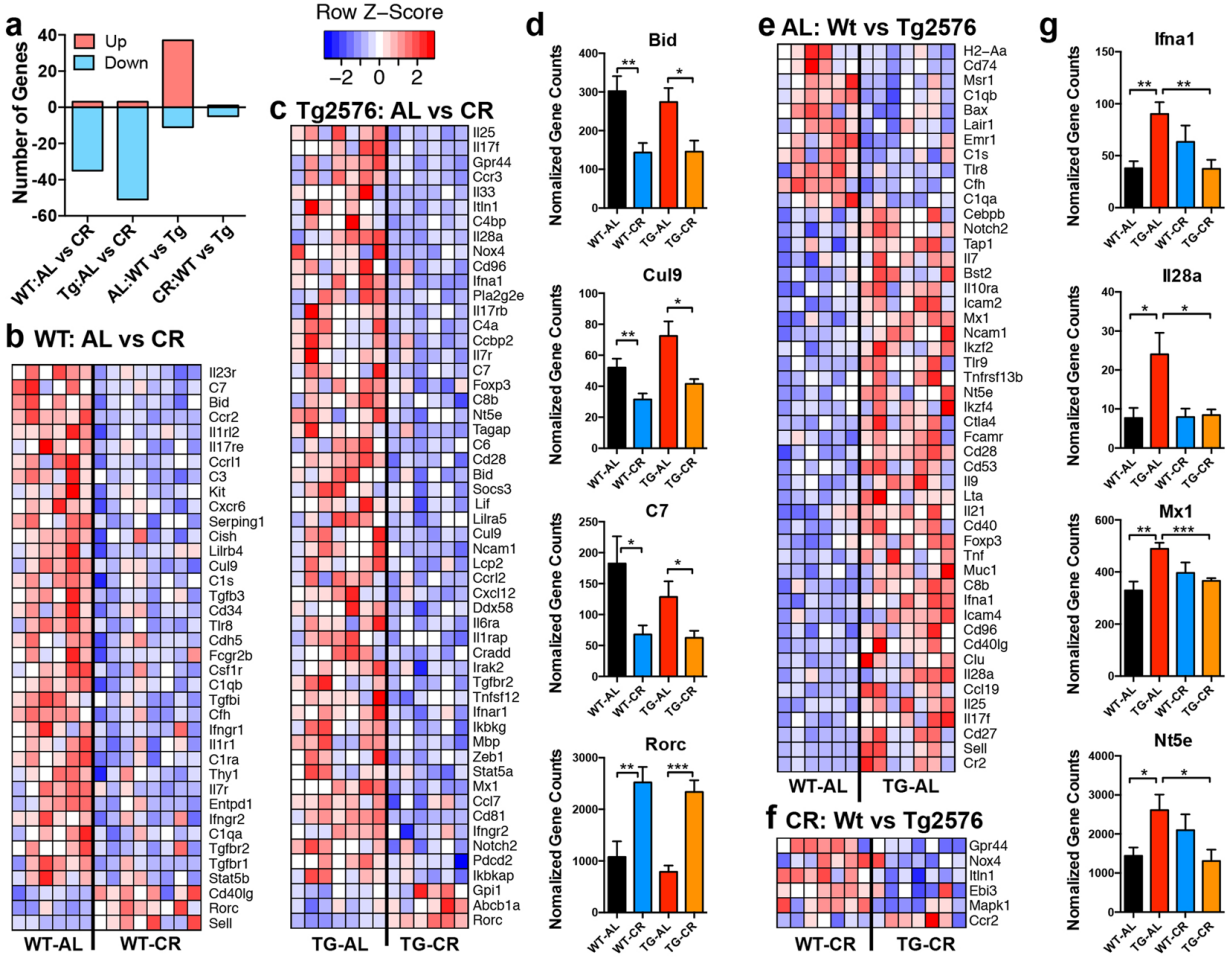
CR reverses APP-associated gene expression in the intestine. Ileal gene expression was measured by Nanostring nCounter analysis in 15 MO female mice. (**a**) Enumeration of significantly upregulated or downregulated genes modulated by diet in WT or Tg2576 mice and modulated by genotype in AL- or CR-fed mice. (**b**,**c**) Expression levels of genes that are modulated by diet in WT (**b**) and in Tg2576 (**c**) mice. (**d**) Selection of genes altered by diet in both WT and Tg2576 mice. (**e**,**f**) Expression levels of genes that differ between WT and Tg2576 littermates in AL (**e**) and in CR (**f**) mice. (**g**) Selection of genes that show altered expression in AL-fed Tg2576 mice compared to AL-fed WT mice, which are reduced to WT levels with a CR diet. *p < 0.05, **p < 0.01, ***p < 0.001 t-test.

We sought to characterize the global effects of CR on gut immune responses, as this could be an intermediary between the microbiota and the brain. We measured gene expression in the ileum of 15 MO WT and Tg2576 female mice using the nCounter Nanostring Immunology Panel of 547 genes related to inflammation and immune function. Results revealed that CR disproportionally led to downregulation in intestinal expression of those genes in both WT (35 genes) and Tg2576 female mice (51 genes), with only 3 genes upregulated **(**Fig. 5a–c**)**. In both WT and Tg2576 mice, the pro-apoptotic genes BH3 interacting-domain death agonist (*Bid)* and cullin-9 *(Cul9)* were downregulated, as well as the membrane attack complex component: complement factor 7 (*C7)*, which promotes programmed cell death via a Bid-dependent pathway^31^ **(**Fig. 5d**)**. CR also upregulated retinoic alpha-related orphan receptor c (*Rorc)*, the transcription factor for Th17 cells, which contributes to defense against intestinal pathogens^32^. Examining the effect of APPswe expression, there were multiple genes that were significantly altered between AL-fed Tg2576 mice and WT mice (37 upregulated and 11 downregulated), whereas only 6 genes were altered in parallel between CR-fed Tg576 and WT mice (1 upregulated and 5 downregulated), consistent with the hypothesis that CR reverses APP-driven changes within the intestine in aged mice **(**Fig. 5e,f**)**. Several genes increased in AL-fed Tg2576 mice compared to AL-fed WT mice were reduced by CR, including antiviral responsive genes interferon alpha1 (*Ifna1)*, interleukin-28a (*IL28a*, also known as interferon lambda 2), and Myxovirus resistance 1 (*Mx1)* **(**Fig. 5g**)**. Ecto-5′-nucleotidase (*NT5E)*, which converts adenosine-monophosphate into adenosine^33^, was also elevated in AL-fed Tg2576 mice, and reduced by CR. In summary, we demonstrate for the first time that an animal model of AD shows an inflammatory intestinal transcriptomic profile compared to WT mice, and that CR can reverse this effect.

We also examined transcriptional changes in male mice **(**Supplementary Fig. 6**)**. Consistent with the effects observed in females, CR upregulated T cell transcription factor *Rorc* and downregulated the pro-apoptotic gene *Bid*. Unlike CR-fed female mice who showed a global decrease in intestinal expression of immune genes, CR-fed males had a similar number of upregulated and downregulated genes. Examining differences related to APP, Tg2576 males had many genes that were downregulated compared to WT, including T-cell markers, *Cd3e*, and *Ccr9*, which plays a role in T and plasma cell homing to the gut. Unlike females, CR in males did not reverse AD-related transcriptional changes in the gut.

### *B. fragilis* increases Aβ plaque deposition in female mice

**Figure 6:**
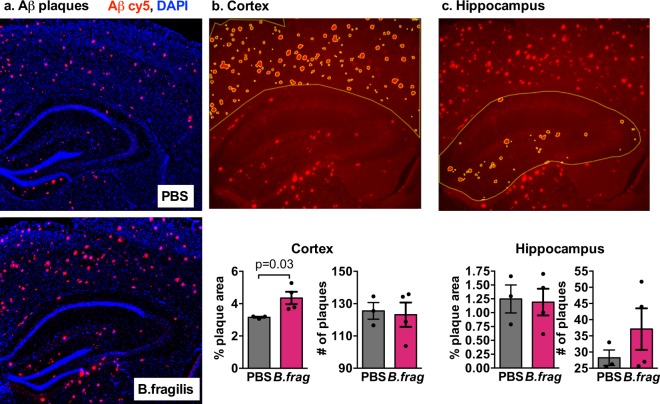
*Bacteroides fragilis* promotes Aβ deposition. (**a**) Merged fluorescent image of Aβ (red) and nuclei (blue). (**b**,**c**) Cortical (**b**) and hippocampal (**c**) regions were outlined in 3 brain sections per mouse and plaque area and number were quantified in ImageJ. Aβ-labeled plaques are shown in red, region of interest and automatically detected plaques shown in yellow. p-value listed for Student t-test, n = 3–4 mice/group.

Because *Bacteroides* showed the greatest abundance increase in aging in the AL-fed Tg2576 females, which we linked with Aβ plaques, we tested whether *B. fragilis* could drive plaque deposition *in vivo*. We administered the Type strain of *B. fragilis* DSM2151 or pre-reduced anaerobically sterilized (PRAS) saline as control to 2.5 MO APP/PS1 female mice. The 2.5 MO point was selected to accelerate Aβ pathogenesis at the time of disease onset but before cognitive signs would become apparent. We selected the APP/PS1 construct in order to accelerate the histopathological endpoints for AD. These APP/PS1 mice have the same APPswe mutation (KM670/671 NL) as Tg2576 mice, and in addition have the L166P mutation in PSEN1 (encoding a subunit of γ-secretase) under the control of the endogenous Thy1 promoter. These mice begin to show the first signs of Aβ pathology in the cortex at 2 MO and in the hippocampus by 4 MO. We assessed plaque deposition by immunohistochemistry with fluorescent antibodies and found that the mice that received *B. fragilis* had increased plaque size in the cortex **(**Fig. 6**)**. This provides evidence that *B. fragilis*, which is enriched in aging and AD, may contribute to disease pathogenesis.

## Discussion

The composition of the microbiota changes with age in humans^12^ and in relevant animal models^14,34^, consistent with the hypothesis that dysfunction in the microbiome could contribute to age-related diseases, including AD. Lifestyle modifications that reduce the risk of AD, including diet and exercise, can modulate the aging microbiota^7^, which may prove to be major public health prevention strategies. We demonstrate that female mice with hallmark AD lesions develop substantially larger changes in their microbiota with aging, and that age-related changes in the microbiota could be rescued by a CR diet.

We found that *Bacteroides* abundance correlates with aging and plaque pathology. In humans, *Bacteroides* increase with both aging^13^ and AD^6,35^, and their toxins have been hypothesized to play a role in AD^26^. We demonstrate that the Type strain of *Bacteroides fragilis* can increase Aβ plaques in the brain. This is consistent with our hypothesis that beneficial effects female mice receive from CR are partially mediated by controlling age-related changes in the microbiome. *Bacteroides* species have highly varied carbohydrate and polysaccharide utilization genes which are important in the establishment and maintenance of colonization^36,37^. The CR diet limited calories only in carbohydrates, which likely directly impacted *Bacteroides* colonization.

Gut microbiome alterations have been observed in patients with AD^6,7,38^. Levels of *Bacteroides* are elevated in AD and correlate with blood levels of Aβ levels^6,35^. This is consistent with our finding that *Bacteroides* was increased in aging associated with higher plaque burden in females. Paradoxically, some studies have found lower levels of *Bacteroides* in AD patients^7,32^. *Bacteroides* can have differential effects in health and disease, based on strain variation. For example, *B. fragilis* strains producing polysaccharide A induce regulatory T cells, reduce inflammation, and improve disease severity in experimental autoimmune encephalomyelitis, the animal model for multiple sclerosis^39^. In contrast, enterotoxigenic *B. fragilis* strains promote inflammation^40^. Further studies to examine the role of different strains of *Bacteroides* in the pathogenesis of AD are warranted.

Altered microbiota has been observed in several AD animal models^41–47^. Treating APP/PS1 mice with broad spectrum low-dose antibiotics led to decreased Aβ plaque deposition in male but not female mice with the protective effect associated with increased *Allobaculum, Akkermansia*, and *Lachnospiraceae*^9^. In another study, WT mice had higher levels of *Allobaculum* compared to APP/PS1 mice and transfer of WT microbiota decreased Aβ plaques in APP/PS1 mice^8^. The genus *Faecalibaculum*, which we identified as potentially protective, was only discovered from culturing the mouse microbiota in 2015^48^. There is little known about *Faecalibaculum* and it is missing from the commonly used taxonomic database, GreenGenes^49^. When GreenGenes is used for taxonomic assignment, this bacterium is classified as *Allobaculum*, a bacterial taxon originally isolated from a canine^50^. Thus, it is possible that other studies reporting findings on *Allobaculum* could in fact be identifying *Faecalibaculum*. *Allobaculum* has been associated with both increased lifespan in response to CR and improved metabolic health in a model of antibiotic-induced obesity^18,51^, and was depleted in other animal models of AD^9^, consistent with the hypothesis that these are potentially protective bacteria. Taken together, these studies indicate microbiota alterations drive Aβ pathogenesis and suggest that *Faecalibaculum* or closely related species could be key players, which have translational implications.

Altered microbiota composition in aging and AD implies altered microbiota function, which could contribute to AD. We identified microbiota pathways involved in amino acid metabolism, vitamin production, and antioxidant production linked with Aβ levels in the brain that showed enhanced age-related changes in AD mice. This suggests that the AD brain may be more vulnerable to pathogenic fluctuations in the gut microbiota associated with aging, providing a clear role for peripheral changes mediating brain-level alterations in AD. Microbial amino acid metabolism modulates inflammation in multiple sclerosis animal models^30^, and the gut microbiota can alter oxidative stress, known to enhance neurodegeneration in AD^52^. Specifically, we observed an age-related increase in predicted microbial genes involved in isoleucine biosynthesis in Tg2576 mice, which was reversed by CR. It has recently been shown that the sodium oligomannate prevents Aβ accumulation, tau phosphorylation, an cognitive decline in an animal model of AD, which was linked with a reduction in microbially produced isoleucine^53^. Thus, altered microbiota homeostatic metabolism in aging may be one mechanism by which the microbiota contributes to AD.

Another mechanism by which the aging gut microbiota may contribute to AD is by modulating intestinal and systemic immunity. Microbiota from aged mice elevate circulating inflammatory cytokines and impair macrophage phagocytosis when transferred to young mice^14^. Systemic inflammation can contribute to cognitive decline in AD^54^, and depleting the microbiota can modulate immunity in the central nervous system in animal models of AD^8,9,55^. We establish a mechanistic link between AD pathology and intestinal inflammation. Specifically, we find an inflammatory intestinal gene signature in aged Tg2576 female mice compared to WT littermates, which was abolished by CR. In particular, we found increased expression of *Ifna1*, *IL-28a* and their downstream target *Mx1*, which are involved in Th1 inflammatory responses, as well as *Nt5e* (also known as *CD73*), which substantially regulates extracellular adenosine. Since cell injury releases pro-inflammatory ATP, which is then converted by *CD39* to AMP and to (anti-inflammatory) adenosine by *Nt5e*, elevated *Nt5e* levels are a marker of increased tissue damage and inflammation^33^. In the brain, *Nt5e* can activate, rather than repress, microglia in a Parkinson’s disease model and contribute to neuronal toxicity^31^. Centenarian humans have lower *Nt5e* levels compared to octogenarians^56^, suggesting that a physiologic decrease is associated with normal, healthy aging.

Aging leads to a loss of gut barrier function and dysregulation at the mucosal surfaces^57^, which could contribute to AD. Aged microbiota increases both intestinal inflammation including complement activation^34^ and intestinal permeability, which lead to circulating microbial cell wall components and systemic inflammation^14^. CR increases gut barrier function^58^, and reduces serum lipopolysaccharide binding protein in aged mice^18^. Decreased intestinal expression of pro-apoptotic genes *Bid, Cul9*^31^, and complement factor *C7* that we observed in both aged WT and Tg2576 mice, suggestsing that CR may lower inflammation in female mice and increase intestinal cell survival. CR also increased the expression of *Rorc*, the Th17 cell transcription factor, which plays a critical defensive role against intestinal pathogens^32^. Thus, CR appears to modulate intestinal physiology to limit inflammation and maintain defenses in females. In both males and females, CR reduced *Bid* and increased *Rorc*. However, beyond these genes, few changes were conserved. A major sex-specific difference is that AL-fed female Tg2576 mice show increased expression of inflammatory genes compared in WT, which is reversed by CR. In contrast, AL-fed Tg2576 male mice show decreased expression of inflammatory genes compared to WT, which is not altered by CR.

Age-related microbiota changes were more pronounced in Tg2576 females than in males, despite being littermates. AD is more prevalent in females in both humans^59,60^ and in animal AD models^61^. This study of sex-specific microbiota associations in AD models reinforces the importance of studying sex differences, which may have therapeutic implications^24^. CR improves gut barrier function, reduces inflammation, alters the microbiota^18^, and extends lifespan in females^62^. Sexually-dimorphic gastrointestinal tract aging critically mediates the benefits that females derive from this diet^62^. CR also reduces Aβ plaque in Tg2576 females but not males^16^, which we now link to the microbiota. Sex-specific differences in the microbiota can contribute to disease. In the NOD mouse model of type 1 diabetes, females have a higher disease incidence than males, but can be protected when colonized with male microbiota, indicating that sex-dependent changes in the microbiota can drive disease pathology^23^. Furthermore, broad-spectrum antibiotics protect male, but not female mice from Aβ accumulation^9^, and the microbiota modulate transcription in microglia, the key immune cells of the brain, in a sex-dependent manner^63^. We found that *Bacteroides* levels are lower in male than in female mice. Whether this microbiota difference could account for lower plaque load in males or whether *B. fragilis* could drive AD pathogenesis in in males remains to be determined.

A major unanswered question is whether other bacterial species selected by CR or by male sex could have a beneficial effect, thus it would be useful to assess whether transfer of whole microbiota from CR-fed mice or from aged males would decrease plaque load in aged AL- fed females. Long-term exposure to the microbiota in young animals has been achieved by co-housing an aged germ-free mouse with a young conventionally raised mouse^14^. The approach of co-housing males with females or co-housing CR is not feasible in the context of male to female microbiota transfer (due to risk of pregnancy), or in the CR paradigm in which the calories are controlled by providing a daily allotment of food to singly housed mice. However, an alternate microbiota could be transferred between males and females via exposure to bedding materials, or long-term administration of microbiota via oral gavage.

Although CR may be a translationally relevant, nontoxic, and inexpensive modality to prevent AD-like pathology throughout life, there are concerns with nutritive status and frailty in aging that complicate later-life interventions. Evidence from our group and others suggests that CR can prevent or reduce Aβ burden when initiated before overt plaque deposition^17,64,65^ or after plaque pathology has developed^66^. In a translational context, it would be more practical to apply CR at the onset of AD-related pathology to prevent further decline. However, we anticipate that introduction of restricted feeding at late stages of pathology and/or old age may not be universally well-tolerated. When CR is introduced at to mice 17 MO, which is commensurate with the age of emergence of memory deficits in our animal models, it leads to higher mortality, in contrast to lengthening lifespan when initiated in younger mice^67^.

Approaches to treat AD in aging that are microbiota-based may have profound translational utility. Our data show that CR can rescue age- and APP-related microbiome changes, implicating the microbiota as a potential therapeutic target in aging and AD. Sex-specific microbiota interactions that we and others observe with AD suggests that separate therapies may be needed for males and females. Whether or not the protective effects of CR are microbiota-mediated and whether there are similar relationships between the microbiota and tau pathology remains unknown. Although this study identifies key relationships between the microbiome and AD-related Aβ pathogenesis in aging that can be reversed by diet, applications to humans will require much further study and limitations may exist. Currently, most available probiotics contain *Lactobacillus*, *Bifidobacterium*, and *Streptococcus*, but administration of *Lactobacillus* and *Bifidobacterium* had no effect on cognitive function in AD patients^68^, suggesting that new generation probiotics with AD-specific indications remains a future approach.

## Materials and Methods

### Animal care

Animal protocols were approved by the Institutional Animal Care and Use Committee (IACUC) of the Nathan Kline Institute/NYU Langone Medical Center and by the IACUC at the Brigham & Women’s Hospital and were in full accordance with NIH guidelines. Tg2576 mice, which harbor a human variant of APP termed the “Swedish” mutation (APPswe), (Lys670/Asn and Met671/Leu), and nontransgenic littermates on a Swiss Webster DBA/C57BL6 F1 background were used for CR studies. APP/PS1 mice were used for the *Bacteroides fragilis* experiments. Mice were kept on a 12-hour light-dark cycle in temperature-controlled conditions.

### CR feeding regimen

At approximately 2.5 MO, male and female, WT and Tg2576 littermates (n = 15–17 per group) were randomized to either an AL or CR diet. Mice were singly housed in order to ensure that the appropriate number of calories were consumed. For every 1 g consumed by an AL-fed animal, 0.71 g of the CR diet was administered to the CR cohort. The AL and CR diets were prepared by Research Diets (New Brunswick, NJ), and were designed with matched protein and fat levels, with a 30% reduction in carbohydrates only as previously described^17^. Approximately half of each group was sacrificed at 5.6 MO and the other half was sacrificed at 15 MO. For the aged-cohort we employed AL-fed WT mice (n = 9 females and 8 males), CR-fed WT mice (n = 9 females and 8 males), AL-fed Tg2576 mice n = 7 females and 8 males, CR-fed WT mice (n = 8 females and 8 males).

### Assessment of Aβ plaque deposition in Tg2576 mice

At approximately 15 MO, mice were euthanized with an overdose of ketamine and xylazine and perfused transcardially with ice-cold 0.1M phosphate buffer. Brains were rapidly removed and 1 hemibrain was used for histopathologic analysis of human Aβ as previously described^17^. The entorhinal cortex and the hippocampus were dissected from the other hemibrain, and used for measurement of Aβ40 and Aβ42 protein levels quantified via colorimetric sandwich ELISA as previously described^17^. The data presented in this manuscript regarding the effect of CR on Aβ plaque load **(**Fig. 3a–c**)** is a new representation of findings originally published in Neurobiology of Aging, Schafer M. J. *et al*., Reduction of beta-amyloid and gamma-secretase by calorie restriction in female Tg2576 mice, 36:1293–1302, Copyright Elsevier (2015)^17^. Published with permission from Elsevier.

### 16S microbiota sequencing and analysis

Fecal microbiota samples were collected from WT and Tg2576 mice prior to dietary intervention (study day 0), then 1 day, 1 week, 1 month, and approximately every two months thereafter, and stored at −80 °C. DNA was extracted using the MoBio PowerLyzer kit. The V4 region of the 16S rRNA gene was amplified with barcoded-fusion primers and paired-end 151 base-pair reads were sequenced on the Illumina MiSeq instrument as previously described^51,69^. Quantitative insights for microbial ecology (QIIME) was used for quality filtering and downstream analysis for β-diversity, and compositional analysis^70^. Sequences were filtered for quality by trimming reads below a quality score of q20 and discarding reads shorter than 75% percent of the original length. OTUs were picked using the de-novo method and taxonomy was assigned using the Silva 132 database^71^. Distances between samples (β-diversity), were calculated using the phylogenetic based distance UniFrac^72,73^. Statistical testing for differential clustering of samples on the PCoA plots was performed using the Permanova test using 999 permutations. Significant differences in taxa modulated by genotype and diet was determined by linear discriminant analysis effect size (LEfSe)^74^. Metagenomic content of the microbiota samples was predicted from the 16S rRNA profiles and KEGG pathway functions were categorized at level 3 using the phylogenetic investigation of communities by reconstruction of unobserved states (PICRUSt) tool^27^. Operational taxonomic units (OTUs) or predicted KEGG metagenomic pathways associated with Aβ40 or Aβ42 levels in the hippocampus were identified using a random forest model and the Boruta algorithm^25^. Spearman correlations between microbial abundance and Aβ40 and Aβ42 levels was calculated in the R statistical framework^75^ using the cor.test in the stats package and plotted using the ellipse package^76^.

### Intestinal transcriptomic profiling

Distal ileum samples were collected from 15 MO female mice, microbiota contents were removed by gentle scraping. Methods were taken to prevent RNA degradation, including using methods for inactivating RNAses on laboratory instruments and surfaces using RNAse-ZAP (ThermoFisher). Samples also were preserved in RNAlater to block the activity of tissue RNAses. RNA was extracted using the Qiagen RNeasy kit. We previously validated that our methods yielded intact RNA, as visualized as two distinct bands on a 3-[N-morpholino]propane sulfonic acid (MOPS) gel. In this study, we did not directly measure the RNA integrity number (RIN) on the bioanalyzer chip, however, our work in the past yielded high quality RNA with an average RIN of 9^16^. We further assessed RNA quality using Nanodrop. Samples with a low 260/280 ratio indicating protein contamination, or a low 260/230 ratio indicating solvent contamination, underwent further purification using ethanol precipitation. 100 ng RNA from each sample was analyzed on the Nanostring Immunology Panel, consisting of 547 genes. Transcriptional profiles were normalized using the nSolver software using the default (*i*) background subtraction of the geometric mean of negative controls, (*ii*) normalization by positive controls, and (*iii*) housekeeping genes including on the Nanostring Panel. Low abundance genes with an average count less than 10 were excluded from analysis, and differential testing was performed using Student’s T-test. Heat-maps were made using R package gplots^77^.

### Assessment of the effect of *B. fragilis* on Aβ plaque deposition

In order to accelerate the histopathological endpoints for AD, we used the APP/PS1 model, which have the same APPswe (KM670/671 NL) mutation in APP as the Tg2576 mice as well as the L166P mutation in PSEN1 (coding for a subunit of γ-secretase), under the control of the endogenous Thy1 promoter^78^. APP/PS1 mice begin to show the first signs of Aβ pathology at 2 MO in the cortex and in the hippocampus by 4 MO^78–81^. 2.5 MO female mice were randomly assigned to receive *B. fragilis* gavage (n = 4) or pre-reduced anaerobically sterilized (PRAS) saline as control (n = 3). This timepoint was selected to accelerate Aβ pathogenesis at the time of disease onset but before cognitive symptoms would become apparent. *B. fragilis* was given once a week for 10 weeks. Mice were housed in sterilized micro-isolator cages in a biosafety level 2 (BSL2) facility and AL-fed on standard chow. All animal experimental procedures were performed in accordance with the approved Animal Care and Use Protocols and began when the animals were approximately 10 weeks.

*B. fragilis* strain DSM2151 (corresponding to ATCC strain 25285) was obtained from the DSMZ-German Collection of Microorganisms. This strain was selected because it is the *Type* strain, the first strain of *B. fragilis*. This strain was originally isolated in 1898 from an appendix abscess^82^. Bacteria were grown for 48 h on Brucella agar plates with sheep blood, vitamin K and hemin in an anaerobic chamber, and sub-cultured in between to maintain a fresh culture with high viability. The gavage of the mice was performed 3 times during the 1^st^ week and then once a week for 9 weeks. Each mouse received 0.2 mL of PBS or of *B. fragilis* inoculum with a turbidity of 0.70 ± 0.1 optical density (OD) units, which corresponds to 1 × 10^9^ bacteria/mL.

At 5 MO, mice were euthanized with CO2 narcosis and perfused transcardially with ice-cold HBSS. Brains were rapidly removed and the left hemibrain was fixed for 24 h in 4% Paraformaldehyde (PFA), followed by an immersion in a 30% sucrose solution until they were no longer buoyant. The brain was then frozen in OCT in a dry-ice ethanol bath, and kept at −30 °C. The hemispheres were then sliced coronally with a cryostat with a width of 20 microns. After drying, the slices were kept at −30 °C. Just before staining, tissue sections were thawed and fixed with acetone. Sections were incubation with blocking buffer (10% Normal Horse Serum +2% Bovine Serum Albumin +1% glycerin +0.1% Triton X-100 in PBS), washed, and incubated overnight with at 1:300 dilution of Purified anti-β-Amyloid, 1–16 Antibody, clone 6E10 (Biolegend catalog 803001), which recognizes amino acids 3–8 of Aβ and APP. The next day, the slides were washed again, incubated with the secondary antibody Cy5 AffiniPure Goat Anti-Mouse IgG, Fcγ subclass 1 specific, (Jackson Immuno Research catalog 115-175-205) at a 1:300 dilution. Then washed, dried, and the coverslip was attached with VECTASHIELD Antifade Mounting Medium with DAPI (Vector Laboratories). After drying overnight at room temperature in the dark, slides were kept at 4 °C (then at −30 °C for long term storage). The images were taken with fluorescent Leica microscope, with 5x magnification, an exposure of 300 ms and a light intensity of 81% for DAPI and of 100% for the Cy5 channel. Using ImageJ, the hippocampus and the overlying cortex above it were analyzed separately. The background was subtracted (0.30) and the threshold was calculated automatically using the triangle algorithm. The percent area covered by the plaques within the traced region of interest (cortex or hippocampus) was calculated, and differences between *B. fragilis* and control were determined by t-test.

## Supplementary information

Supplementary Information

## Data Availability

Sequencing data from the microbiota 16S rRNA is submitted in the NCBI short-read archive under Bioproject number PRJNA573528.
