# Supplementary material for: Calorie restriction slows age-related microbiota changes in an Alzheimer’s disease model in female mice
Source: Sci Rep. 2019 Nov 29;9:17904. doi: 10.1038/s41598-019-54187-x (PMC6884494; doi:10.1038/s41598-019-54187-x)
Supplement: Supplementary file 1 — Supplementary Information [file 41598_2019_54187_MOESM1_ESM.pdf]

Supplementary Information

Calorie restriction slows age-related microbiota changes in an Alzheimer's disease model in female mice

Laura M. Cox[1,2], Marissa J. Schafer[3,4,5,6], Jiho Sohn[2,7], Julia Vincentini,[8] Howard L. Weiner[1], Stephen D. Ginsberg[4,5], Martin J. Blaser[2,9]

[1] Ann Romney Center for Neurologic Diseases, Brigham & Women's Hospital, Harvard Medical School, Boston, MA, USA
[2] Department of Medicine, NYU Langone Medical Center, New York, NY, USA
[3] Cellular and Molecular Biology Training Program, NYU Langone Medical Center, New York, NY, USA
[4] Psychiatry, Neuroscience & Physiology & the NYU Neuroscience Institute, NYU Langone Medical Center, New York, NY, USA
[5] Center for Dementia Research, Nathan Kline Institute, Orangeburg, NY, USA
[6] Department of Physical Medicine and Rehabilitation and Robert and Arlene Kogod Center on Aging, Mayo Clinic, Rochester, MN, US
[7] Jacobs School of Medicine and Biomedical Sciences, University at Buffalo, State University of New York, Buffalo, NY, USA
[8] Ecole Polytechnique Fédérale de Lausanne (EPFL), Lausanne, Switzerland.
[9] Center for Advanced Biotechnology and Medicine, Rutgers University, New Brunswick NJ, USA

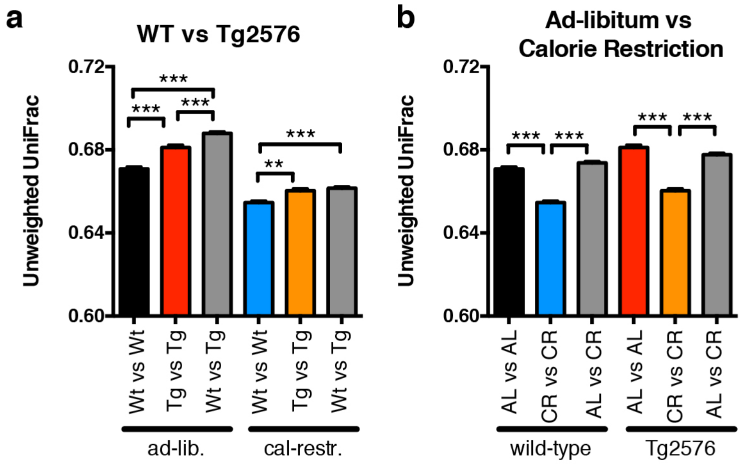

Supplementary Figure 1. APP overexpression and diet shape the microbiota in male mice. Differences between microbiota compositions was assessed by inter- and intra-group unweighted UniFrac distances in male mice. **a)** Similar to females, intergroup UniFrac distances (WT vs. TG, gray bars) are larger than WT or TG intragroup distances. **b)** CR reduces intragroup microbiota variation compared to AL fed mice. Bonferroni adjusted t-test, * $p < 0.05$, ***$p < 0.001$.

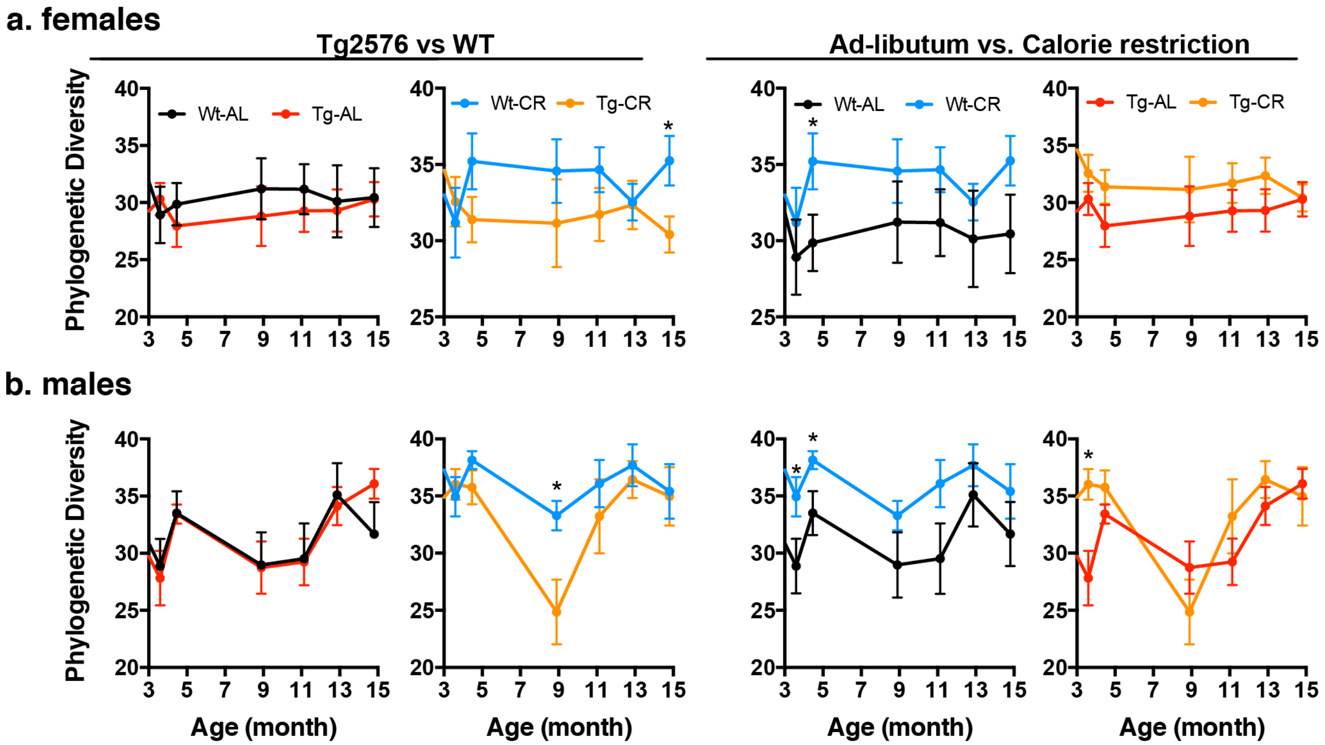

Supplementary Figure 2. The effect of diet and Aβ overexpression on α-diversity. Phylogenetic diversity was measured at an even sampling depth of 5,000 reads per sample over 12 months in WT and Tg2676 mice fed AL or CR diet in female **(a)** and male **(b)** mice. t-test, * $p < 0.05$.

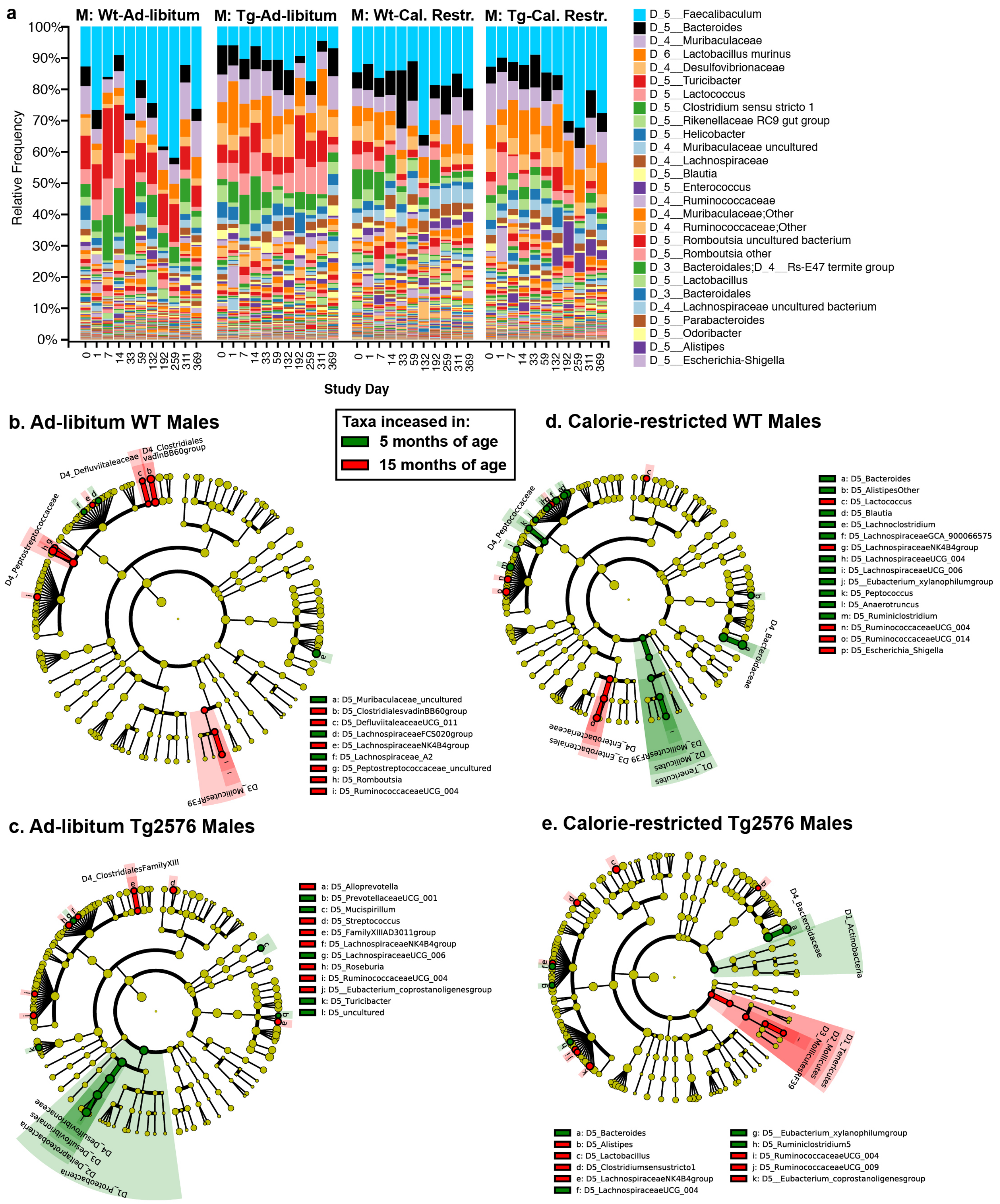

Supplementary Figure 3. The effect of CR on the aging microbiota in male mice. a) Composition of the fecal microbiota from study day 0 (~3 MO) until study day 369 (~15 MO). **b-e)** Microbiota that are increased in 5 MO or 15 MO, LEfSe $p<0.05$. Each cladogram represents all microbiota detected at >0.1%, shown at the Kingdom phylogenetic level through the genus level. A yellow circle depicts taxa present, but not enriched. Red circles are enriched in aging, and green enriched in young animals. The size of the circle corresponds to the population of each taxon.

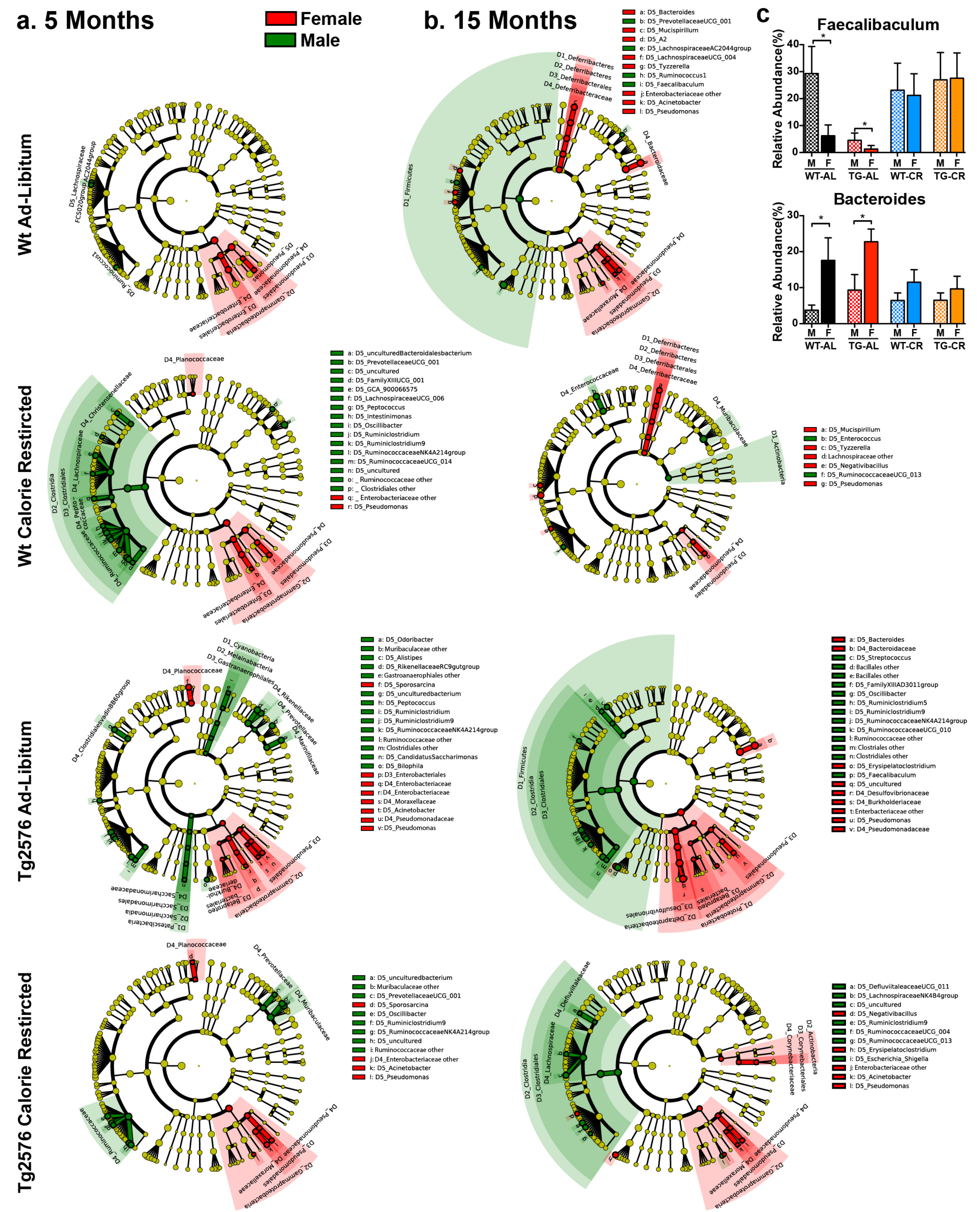

Supplementary Figure 4. Sex-specific differences in the aging and AD microbiota. a-b) Compositional differences between males and females at 5 MO **(a)** and 15 MO **(b)**, LEfSe $p<0.05$. **c)** Relative abundance of *Faecalibaculum* and *Bacteroides* at 15 MO.

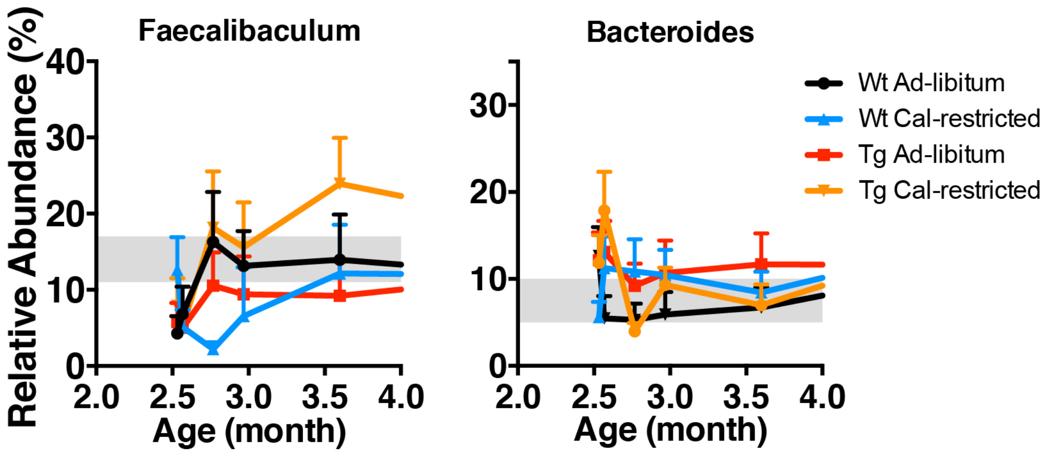

Supplementary Figure 5. Relative abundance of *Faecalibaculum* and *Bacteroides* in the first month of the study. WT and Tg2576 mice were randomly assigned to treatment group, before knowledge of their microbiota composition. During the month following study commencement (2.5-3.5 MO), *Faecalibaculum* levels fluctuated as the mice responded to either calorie-restriction or the control diet. Mice were sampled at baseline, 1 day, 1 week, 2 weeks, and 1 month following dietary intervention. While no change was observed the first day, *Faecalibaculum* levels rose in all groups in the first month on the new diets, with the greatest increases in calorie-restricted Tg2576 mice. *Faecalibaculum* levels eventually declined 10-fold in AL-fed Tg2576 mice. *Bacteroides* did not differ at baseline and showed little fluctuations during the first month. In aging, *Bacteroides* levels increased in AL-fed animals.

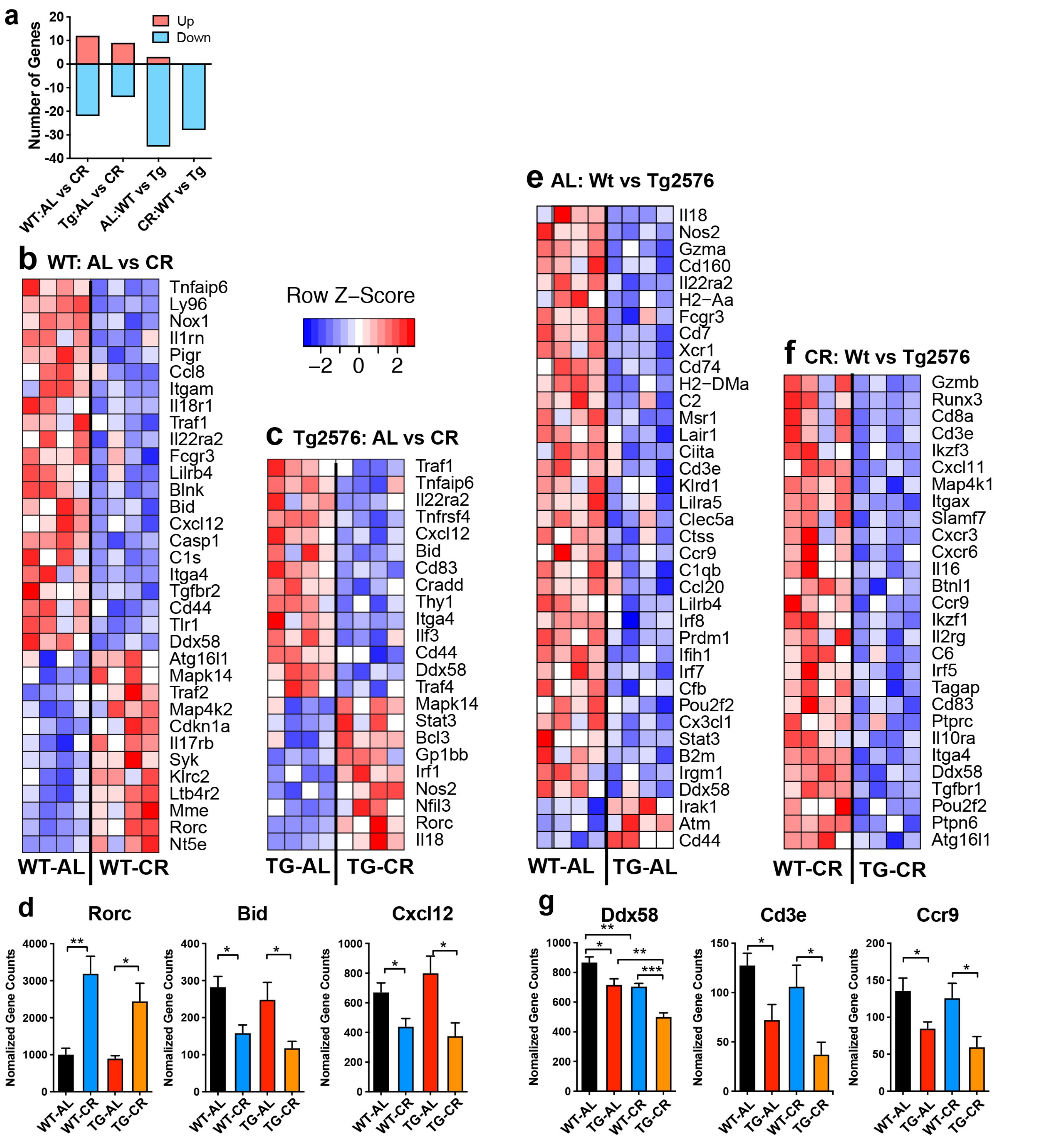

Supplementary Figure 6. Intestinal transcriptional responses to calorie restriction in WT and Tg2576 mice. Ileal gene expression was measured by Nanostring nCounter analysis in 15 MO male mice. **a)** Enumeration of significantly upregulated or downregulated genes modulated by diet in WT or Tg2576 mice and modulated by genotype in AL or CR mice. **b-c)** Expression levels of genes that are modulated by diet in WT **(b)** and in Tg2576 **(c)** mice. **d)** Selection of genes altered by diet in both WT and Tg2576 mice. **e-f)** Expression levels of genes that differ between WT and Tg2576 littermates in AL **(e)** and in CR **(f)** mice. **g)** Selection of genes that show altered expression in AL-fed Tg2576 mice compared to AL-fed WT mice, which are reduced to WT levels with a CR diet. * $p < 0.05$, ** $p < 0.01$, *** $p < 0.001$ t-test.
